# Safety Assessment of a *Periploca chevalieri* Browicz Standardized Extract: Oral Toxicity and Genotoxicity Studies

**DOI:** 10.3390/jox16060198

**Published:** 2026-10-16

**Authors:** Katelene Lima, Maryam Malmir, João Rocha, Rui Pinto, Maria Eduardo Figueira, Maria Paula Duarte, Olga Silva

**Affiliations:** 1 Research Institute for Medicines (iMed.ULisboa), Faculty of Pharmacy, Universidade de Lisboa, 1649-003 Lisboa, Portugal; k.lima@edu.ulisboa.pt (K.L.); m.malmir@edu.ulisboa.pt (M.M.); jrocha@ff.ulisboa.pt (J.R.); rapinto@ff.ulisboa.pt (R.P.); efigueira@ff.ulisboa.pt (M.E.F.); 2 Joaquim Chaves Laboratório de Análises Clínicas, 2790-224 Carnaxide, Portugal; 3 The Mechanical Engineering and Resource Sustainability Center (MEtRICs), Chemistry Department, Nova School of Science and Technology, Universidade Nova de Lisboa, 2829-516 Caparica, Portugal; mpcd@fct.unl.pt

**Keywords:** herbal medicines, *Periploca chevalieri*, safety, oral toxicity, genotoxicity

## Abstract

*Periploca chevalieri* (*Apocynaceae* Juss.) is an endemic medicinal plant from Cabo Verde that has long been used in traditional medicine, particularly for the management of diabetes and cough. However, to date, no studies focusing on potential adverse effects compromising its safe use have been performed. The present study aimed to investigate the safety profile of a standardized 70% hydroethanolic extract of *Periploca chevalieri* aerial parts (PcEE) using complementary in vivo and in vitro toxicological approaches. Repeated oral toxicity of PcEE at doses of 170, 600, and 1000 mg/kg body weight was evaluated in CD-1 mice over 14 days. Clinical signs, body weight, and food intake were monitored, as well as important biochemical parameters (hepatic, renal, and pancreatic biomarkers). Genotoxicity was investigated using the bacterial reverse mutation assay (Ames test) with five different strains of *Salmonella* Typhimurium in the presence and absence of a metabolizing system. The results obtained showed no significant variations (*p* < 0.05) in body weight of the animals treated with PcEE at all tested doses. Significant changes (*p* < 0.05) with negligible relevance were observed in the biochemical parameters of PcEE-treated groups compared to the control group. The Ames test results showed that PcEE did not produce genotoxic effects in any of the tested strains at concentrations up to the maximum tested concentration (5 mg/plate). The results obtained are an important step towards the support and validation of the use of *P. chevalieri* aerial parts in traditional medicine.

## 1. Introduction

Herbal medicines use across the world is mainly supported by traditional knowledge that has been accumulated over centuries and by the documented history of therapeutic application [1,2]. This long history of use has contributed to the common perception that herbal medicines are inherently safe and without adverse effects. In fact, most medicinal plants have been used without being the subject of pharmacological and toxicological assessments, as their efficacy and safety are often inferred from their prolonged traditional use [3].

However, growing scientific evidence has challenged the assumption that herbal medicines are intrinsically harmless [4]. Several studies have reported gastrointestinal disturbances, hepatotoxicity, and nephrotoxicity associated with the chronic use of herbal medicines [5,6,7,8], and, more recently, genotoxic, mutagenic, or carcinogenic risks have also been reported [9,10,11,12].

In line with the mounting evidence on the limitations of relying solely on traditional knowledge as evidence of safety, regulatory authorities such as the OECD (Organization for Economic Cooperation and Development), ICH (International Conference on Harmonization), and EMA (European Medicines Agency) have issued scientific guidelines to oversee the quality and safety of herbal medicines.

Worldwide, there are significant differences related to the legal and regulatory framework for herbal medicines. In several countries, including the United States of America, several European and Asian countries, and some African countries, there are dedicated regulations and guidelines in place to oversee the manufacturing, marketing, and use of herbal medicines [4,13]. However, several African countries still lack an adequate regulatory framework to guarantee the quality and safety of herbal medicines [14,15]. In Cabo Verde, for instance, there are no official legislative or regulatory rules controlling the practice of traditional medicine [14,16], despite the fact that the use of medicinal plants continues to play a key role in primary health care [17,18]. *Periploca chevalieri* (*P. chevalieri*) is an endemic species of the Cabo Verde archipelago (West Africa, ca. 500 km from Senegal) belonging to the genus *Periploca* Tourn. Ex L. and family *Apocynaceae* [19].

The family *Apocynaceae* is well-known for the pharmacological and toxicological significance of its members. While several members of this family are valued for timber and ornamental purposes, others are used as food by certain tribes or as a source of potent poisons [20,21]. *Apocynaceae* family is particularly rich in bioactive secondary metabolites, including cardiac glycosides, alkaloids, terpenoids, steroids, simple phenolic compounds, and flavonoids [20]. Several members of this family are known for their pharmacological potential and toxicity due to the presence of specific alkaloids and/or cardiac glycosides, namely *Nerium oleander* L. [22,23], *Catharanthus roseus* (L.) G. Don [24], *Cascabela thevetia* (L.) Lippold, *Cerbera odollam* Gaertn. [25], *Thevetia peruviana* (Pers.) K. Schum. [22], and some species of the genus *Periploca* [26].

In the genus *Periploca*, some species are characterized as toxic medicinal plants due to the presence of cardiac glycosides [26]. *Periploca sepium* Bge. and *Periploca forrestii* Schltr. are well-known for containing cardiac glycosides such as periplocin and periplogenin, which have therapeutic applications but also carry risks at certain doses, causing cardiotoxicity and hepatotoxicity [27].

*P. chevalieri*, known as “curtecabra”/”lantisco”, is the only representative of the genus *Periploca* in Cabo Verde, where it is used mainly in traditional medicine to treat cough and diabetes [18,28].

Previous studies by our group reported the chemical profile of aqueous and hydroethanolic extracts of *P. chevalieri* aerial parts. The major marker compounds identified were quercetin glycosides (quercetin 3-*O*-glucoronide, quercetin 3-*O*-galactoside, and quercetin 3-*O*-rutinoside), caffeoylquinic acids (chlorogenic acid, cryptochlorogenic acid, and neochlorogenic acid), catechin, epicatechin, and several proanthocyanidins. These studies also demonstrated the antioxidant and antimicrobial activities of these extracts [29], as well as their potential to inhibit carbohydrate-hydrolyzing enzymes (α-amylase and α-glucosidase) and reduce postprandial hyperglycemia in vivo, supporting the traditional use of *P. chevalieri* [30,31,32]. However, despite the medicinal value, its safety profile has remained unexplored. Therefore, this study aimed to assess the oral repeated-dose toxicity and mutagenic potential of a standardized *P. chevalieri* 70% hydroethanolic extract in accordance with internationally recognized regulatory guidelines.

## 2. Materials and Methods

### 2.1. Reagents and Bacterial Strains

Ammonium sodium phosphate dibasic tetrahydrate, anhydrous dipotassium hydrogen phosphate, and sodium chloride were purchased from Fluka (Seelze, Germany). Bacto™ agar was purchased from Becton Dickinson & Co. (Sparks, MD, USA). Citric acid monohydrate, disodium hydrogen phosphate dihydrate, and sodium dihydrogen phosphate monohydrate were purchased from Panreac (Barcelona, Spain). 9–Aminoacridine, 2–aminoanthracene, benzo(a)pyrene, D-biotin, D-(+)-glucose monohydrate, glucose-6-phosphate, dimethyl sulfoxide, nicotinamide adenine dinucleotide phosphate, 2–nitrofluorene, and tert-butyl hydroperoxide were purchased from Sigma-Aldrich (St. Louis, MO, USA). L-Histidine monohydrochloride monohydrate was purchased from Merck (Darmstadt, Germany), magnesium sulfate heptahydrate was purchased from LabChem Inc. (Zelienople, PA, USA), nutrient broth nº 2 was purchased from Oxoid (Basingstoke, UK), and sodium azide was purchased from the J.T. Baker Chemical Company (Phillipsburg, NJ, USA). Aroclor 1254-induced rat liver S9 was purchased from Trinova Biochem (GmbH, Giessen, Germany). *Salmonella enterica subsp. enterica* serovar Typhimurium strains TA98, TA100, TA102, and TA1535 were sourced from the Genetic Department of the Nova Medical School of the Universidade NOVA de Lisboa (Portugal) (received from Professor B.N. Ames (Berkeley, CA, USA)). TA1537 (ATCC, NUMBER: 29630™ (LOT: 7405375)) was obtained from Sigma-Aldrich (St. Louis, MO, USA).

Aroclor 1254-induced rat liver S9 was purchased from Trinova Biochem (GmbH, Giessen, Germany). Ultra-pure water from a Milli-Q water purification system (Millipore, Molsheim, France) was used to prepare all the solutions, dilutions, and culture media.

### 2.2. Plant Material Collection, Identification and Extract Preparation

Aerial parts of *Periploca chevalieri* Browicz (Apocynaceae) were collected from the Natural Park of Serra Malagueta, Santiago Island, Cabo Verde. The plant material was identified by Engineer Samuel Gomes, Head of the Environmental Department of Instituto Nacional de Investigação e Desenvolvimento Agrário (INIDA) of Cabo Verde. The species identification was based on macroscopic examination of vegetative and reproductive structures, comparison with existing herbarium specimens, and confirmation using available taxonomic keys for the flora of Cabo Verde. A voucher specimen (042016KL) was preserved at the Herbarium of INIDA, Santiago, Cabo Verde, for future reference.

The hydroethanolic extract (*Periploca chevalieri* hydroethanolic extract (PcEE)) was prepared by maceration with 70% ethanol in a ratio of 1:10 (1 part aerial parts powder to 10 parts solvent at room temperature (25 °C)) for 72 h.

The obtained extract was filtered, evaporated (40 ± 1 °C), and freeze-dried (Heto, LyoLab 3000, Thermo Fisher Scientific, Leicestershire, UK). After the freeze-drying process, the obtained powdered extract (PcEE) was transferred to amber glass vials, weighed to determine extraction yield and drug extract ratio (DER), and stored at room temperature, protected from light and moisture, until use.

Afterward, total phenolic content and chemical profile were determined (according to Lima et al. 2025 [30]), and the extract stability was monitored periodically (every 6 months) by high-performance liquid chromatography (HPLC) during storage.

For the biological assays, PcEE was diluted in the appropriate vehicle (distilled water for animal assays/DMSO 30% for Ames test) to the final concentrations specified in each assay.

### 2.3. Repeated-Dose Toxicity (14 Days)

#### 2.3.1. Dose Selection and Route of Administration

The selected doses of PcEE were based on the following criteria: the low dose (170 mg/kg bw/day) corresponds to the effective dose identified in our previous study on the management of postprandial hyperglycemia in CD1 mice. The high dose (1000 mg/kg bw/day) corresponds to the highest dose recommended by OECD guideline 407 [33]. The mid-dose (600 mg/kg bw/day) was set at approximately the mean value between the low dose and the high dose, rounded to a practical dosing level. Oral gavage was selected as the route of administration, consistent with the intended route of human exposure.

#### 2.3.2. Animals

A total of 32 male CD-1 mice (5 weeks, 25–40 g) obtained from the Institute of Hygiene and Tropical Medicine (IHMT), Universidade NOVA de Lisboa, were used in this study. The animals were acclimatized for seven days and maintained in cages suitable for rodents, under standard conditions of temperature (22 ± 2 °C) and humidity (40–60%) in a 12 h light–dark cycle. Access to water and food was offered ad libitum. All experimental procedures were performed according to ethical principles of animal research and were previously approved by the committee of the Faculty of Pharmacy, Universidade de Lisboa (protocol CEEE-002/16 approved by the Ethics Committee for Animal Experiments (CEEA)). Additionally, all the experiments were performed according to the ARRIVE Guidelines for Reporting Animal Research.

#### 2.3.3. Experimental Protocol

The oral toxicity test was conducted according to the OECD guideline no. 407 [33] with some modifications and according to our previous work [34]. The animals were sorted into four groups (8 animals/group), and every day for 14 days, distilled water or PcEE at doses of 170, 600, and 1000 mg/kg of body weight (Groups 1–4, respectively) was administered at 10 mL/kg once daily via oral gavage.

The animals were monitored daily for clinical signs of toxicity and every two days for body weight and food intake. At the end of the experiment, the animals were anesthetized (ketamine: xylazine (100 mg/kg:10 mg/kg)), and blood was collected via cardiac puncture for subsequent biochemical analyses. Vital organs (liver, kidney, pancreas, and heart) were collected from each animal in the study to compare with the control group’s organs and for further histopathological studies if signs of toxicity were noted.

#### 2.3.4. Biochemical Analysis

Serum samples obtained by centrifuging the blood samples at 4000 rpm for 15 min were used to measure the levels of aspartate aminotransferase (AST), alanine aminotransferase (ALT), urea, creatinine, amylase, lipase, total cholesterol, high-density lipoprotein [HDL–cholesterol], low-density lipoprotein [LDL–cholesterol], very-low-density lipoprotein [VLDL–cholesterol], triglycerides and total lipids. The analytical determinations were made according to the protocols described by Grácio et al. (2021) [35].

### 2.4. In Vitro Assessment of Genotoxicity/Mutagenicity

The genotoxic potential of PcEE was assessed using the bacterial reverse mutation assay (Ames test) with histidine-auxotrophic *Salmonella enterica* serovar Typhimurium strains TA98, TA100, TA 102, TA 1535, and TA1537, using the plate incorporation method with (+S9) and without (−S9) metabolic activation system. The protocol followed the standard Ames protocol [36] with some modifications [37] and the OECD, EMA, and ICH guidelines [38,39,40] as described in our previous work [34,41].

PcEE was dissolved in 30% DMSO (stock solution) and tested at 250, 625, 1250, 2500, 3750, and 5000 µg/plate without S9, and at 625, 1250, 2500, and 5000 µg/plate with S9. These concentrations were selected after preliminary cytotoxicity testing (a sample was considered cytotoxic when thinning of the background lawn was accompanied by a decrease in His^+^ revertants). The metabolic activation mixture (S9 Mix), prepared from rat livers induced with Aroclor 1254 (500 mg/kg), was freshly prepared before each test.

DMSO (30%) was the negative control. Positive controls without metabolic activation were 2-nitrofluorene (TA98, 5 µg/plate), sodium azide (TA100 and TA1535, 1.5 µg/plate), 9-aminoacridine (TA1537, 100 µg/plate), and tert-butyl hydroperoxide (TA102, 50 µg/plate). With metabolic activation, they were 2-aminoanthracene (2 µg/plate for TA98; 10 µg/plate for TA102, TA1535, and TA1537) and benzo[a]pyrene (5 µg/plate for TA100).

A result was considered positive when a reproducible, concentration-related increase in revertants of at least two-fold (TA98, TA100, and TA102) or three-fold (TA1535, TA1537) over the negative control was observed in at least one strain. All assays were performed in triplicate, and results are expressed as mean ± SD revertant colonies per plate.

### 2.5. Data Analysis

The experimental results were presented as the mean ± S.D. or mean ± S.E.M. The statistical analysis was conducted using GraphPad Prism software (version 8.0, GraphPad, La Jolla, CA, USA). One-way ANOVA followed by a Bonferroni’s post hoc test was applied, and a *p*-value less than 0.05 (*p* ≤ 0.05) was considered significant.

## 3. Results

### 3.1. Genuine Drug Extract Ratio and Extract Chemical Standardization

*P. chevalieri* aerial parts powder was extracted with 70% ethanol as the solvent. The extract obtained was subsequently concentrated under reduced pressure and freeze-dried, yielding a greenish-brown powder with a yield of 40.05% *w*/*w* and a genuine drug extract ratio (DER) of 2.9:1. The standardization was carried out based on total phenolic content, determined with the Folin–Ciocalteu method and high-performance liquid chromatography coupled with mass spectrometry (HPLC-MS) in our previous work [30].

The total phenolic content obtained was 415.2 ± 1.1 mg of gallic acid equivalents/g of dry PcEE. The chemical screening identified 20 major marker compounds (Table S1 of Supplementary Materials), namely, quinic acid, gallocatechin, B-type proanthocyanidin dimers and trimers, neochlorogenic acid, epigallocatechin, catechin, cryptochlorogenic acid, chlorogenic acid, epicatechin, quercetin glycosides (quercetin 3-*O*-rutinoside, quercetin 3-*O*-glucoronide, and quercetin 3-*O*-rutinoside), and 1,4-dicaffeoyilquinic acid [30].

### 3.2. Repeated Dose Toxicity (14 Days) Study

During the repeated dose toxicity study, PcEE was administered daily to male CD-1 mice for 14 days. One control group and three treatment groups (D1–D3) were defined (*n* = 8). The treatment groups were administered PcEE at 170 mg/kg body weight (bw) (PcEE-D1), 600 mg/kg bw (PcEE-D2), and 1000 mg/kg bw (PcEE-D3), while the control group was administered distilled water (vehicle).

#### 3.2.1. Clinical Signs

No treatment-related effect was observed on the behavior and the overall health of the experimental animals (Table S2 of Supplementary Materials).

A total of four deaths (*n* = 4; PcEE-D1: *n* = 1; PcEE-D2: *n* = 1; PcEE-D3: *n* = 2) occurred acutely after oral gavage administration. After necropsy, these deaths were interpreted as gavage-related administration errors (accidental pulmonary instillation of liquid).

At the end of the experimental protocol, macroscopic inspection of the animals’ (*n* = 32) vital organs (liver, kidneys, pancreas, and heart) indicated no changes/lesions compared to the animals in the control group.

#### 3.2.2. Body Weight Variation and Food Intake

**Figure 1:**
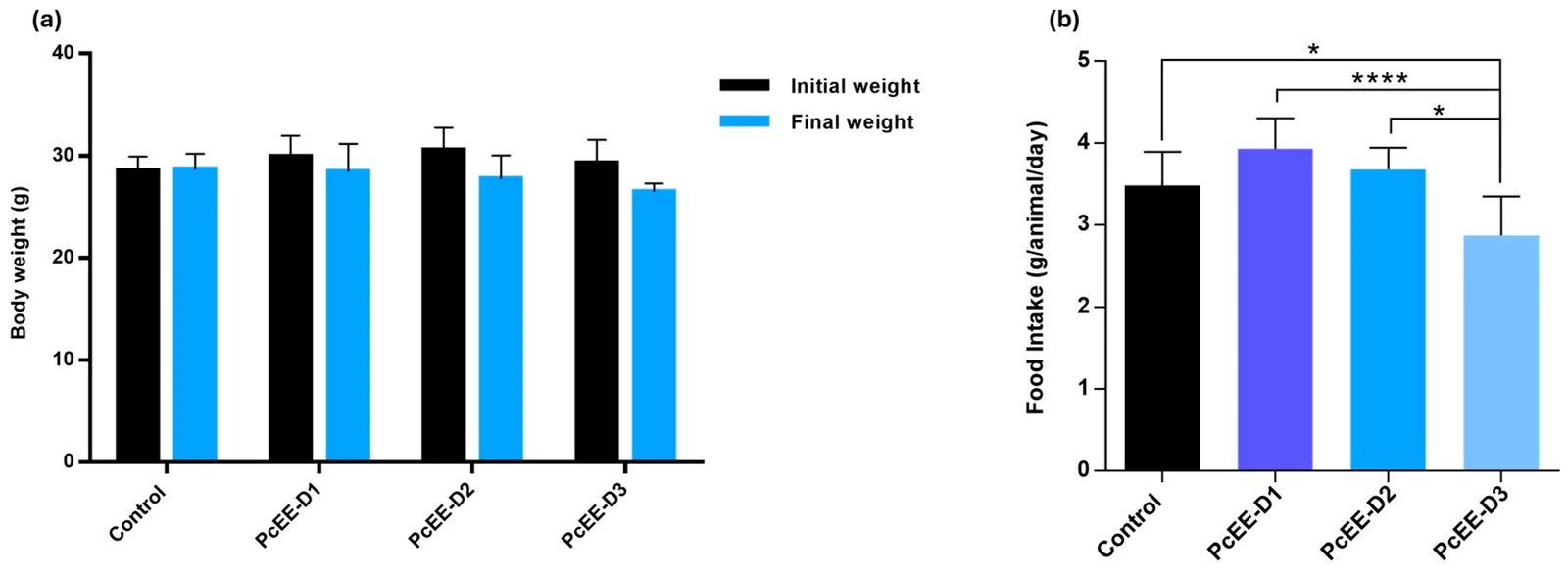
Mean absolute body weight (**a**) and food intake (**b**) of control animals and PcEE-treated animals for 14 days. Data are expressed as the Mean ± S.D., *n* = 6–8. Significance level among different groups: * *p* < 0.05, and **** *p* < 0.0001.

PcEE treatment did not significantly affect the body weight of the treated animals compared to the control group. Sporadic increases and decreases in body weight were observed in all animals during the experimental period; however, overall, no significant differences were observed between their initial and final body weights (Figure 1a).

Regarding food intake, while PcEE-D1- and PcEE-D2-treated groups did not show significant differences in daily food consumption when compared to the control group (Figure 1b), the PcEE-D3-treated group showed a significant decrease in feed intake when compared to the control group (*p* < 0.05) and to PcEE-D1- (*p* < 0.0001) and PcEE-D2 (*p* < 0.05)-treated groups.

#### 3.2.3. Biochemical Parameters

Liver injury biomarkers

**Figure 2:**
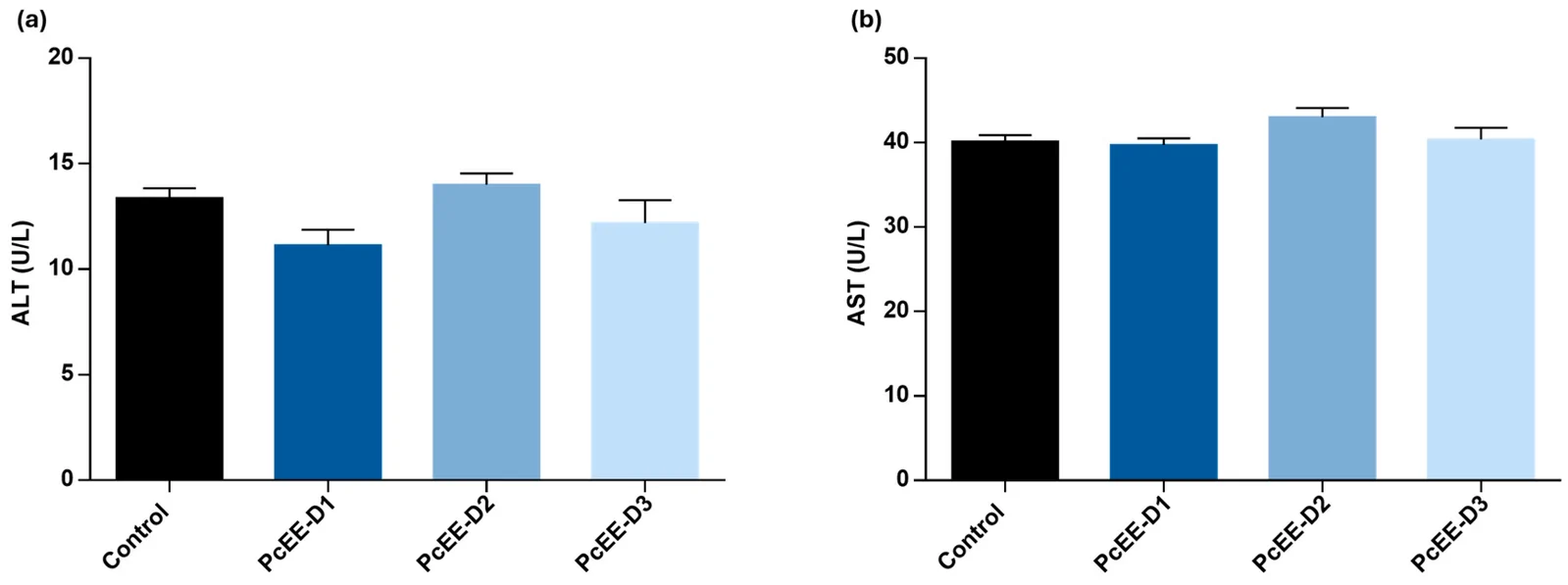
Liver injury biomarkers in CD-1 mice orally administered the hydroethanolic extract of *P. chevalieri* aerial parts (PcEE) at doses of 170, 600, and 1000 mg/kg for 14 days: (**a**) ALT and (**b**) AST and comparison with the control group. Data are expressed as the Mean ± S.E.M., *n* = 6–8. Significance level among different groups.

Hepatic injury was assessed by quantifying the serum biomarkers, alanine aminotransferase (ALT) and aspartate aminotransferase (AST), of all control animals and PcEE-treated animals (Figure 2). The results obtained showed no significant alterations in either ALT or AST across all PcEE-treated groups compared to the control group.

Renal functional biomarkers

**Figure 3:**
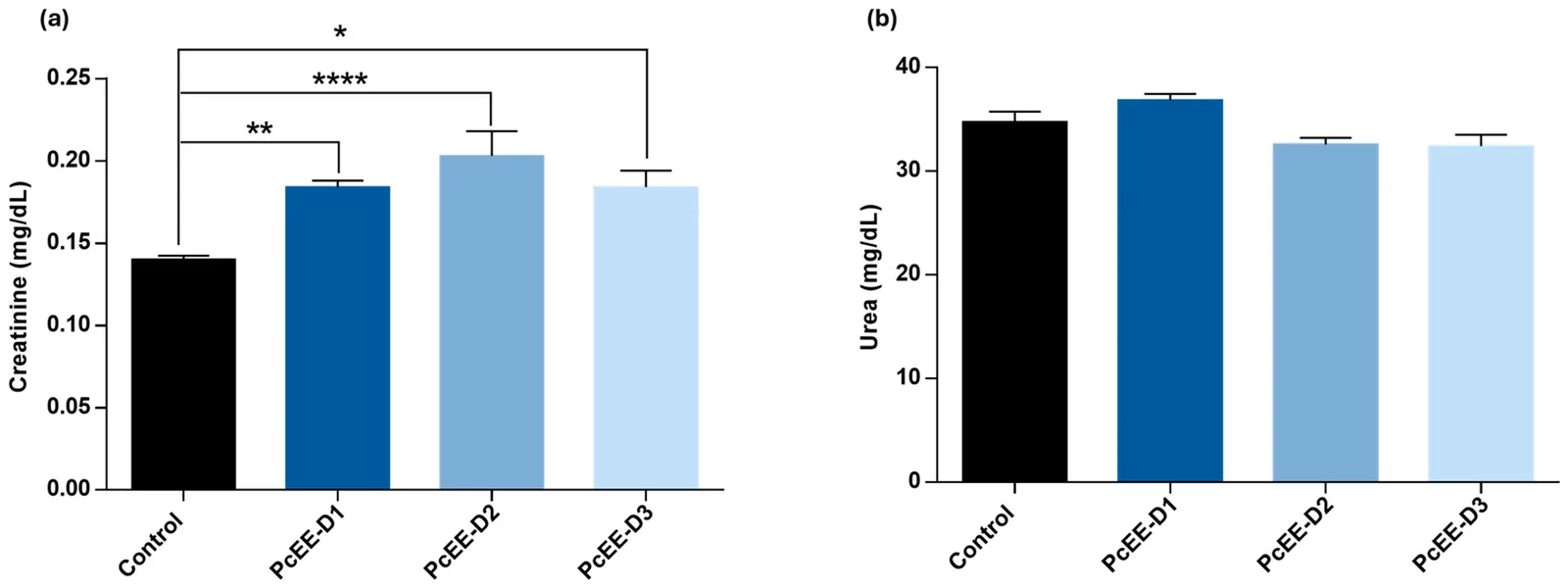
Renal function biomarkers in CD-1 mice orally administered the hydroethanolic extract of *P. chevalieri* aerial parts (PcEE) at doses of 170, 600, and 1000 mg/kg for 14 days: (**a**) creatinine and (**b**) urea and comparison with the control group. Data are expressed as the Mean ± S.E.M., *n* = 6–8. Significance level among different groups: * *p* < 0.05; ** *p* < 0.01; and **** *p* < 0.0001.

The effect of PcEE on renal function was assessed through quantification of serum creatinine and urea concentrations (Figure 3) in all experimental animals. A statistically significant increase in creatinine levels was observed in all PcEE-treated groups when compared to the control group. The highest elevation was observed in the PcEE-D2 group (600 mg/kg) (*p* < 0.0001). Regarding the levels of urea, all the groups in the study revealed similar values, and no significant differences between them were observed.

Pancreatic functional biomarkers

**Figure 4:**
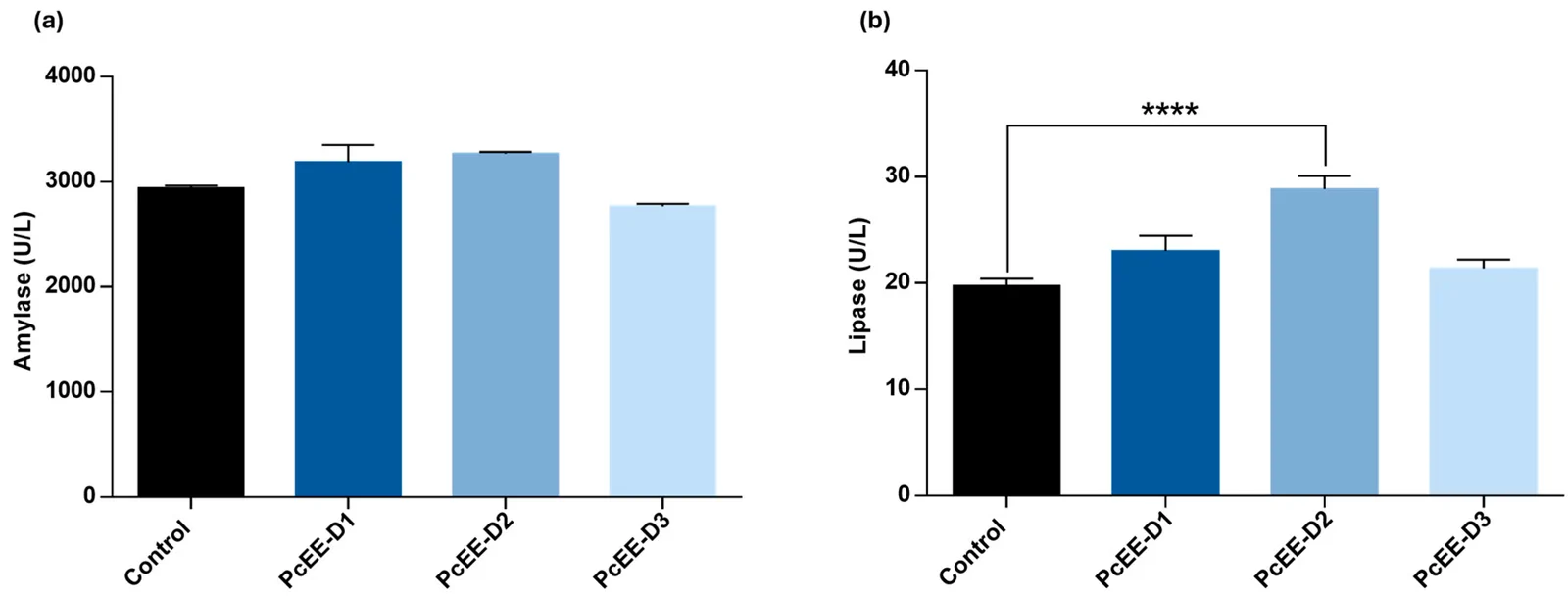
Pancreatic injury biomarkers in CD-1 mice orally administered the hydroethanolic extract of *P. chevalieri* aerial parts (PcEE) at doses of 170, 600, and 1000 mg/kg for 14 days: (**a**) amylase and (**b**) lipase compared with the control group. Data are expressed as the Mean ± S.E.M. *n* = 6–8. Significance level among different groups: **** *p* < 0.0001.

Pancreatic function was evaluated through serum amylase and lipase quantification (Figure 4). The results showed similar levels of amylase between all groups. Regarding the lipase biomarker, while the PcEE-D1- and PcEE-D3-treated groups showed similar levels relative to the control group, PcEE-D3 (600 mg/kg) showed an increase in lipase with statistical significance (*p* < 0.0001).

Lipid profile biomarkers

**Figure 5:**
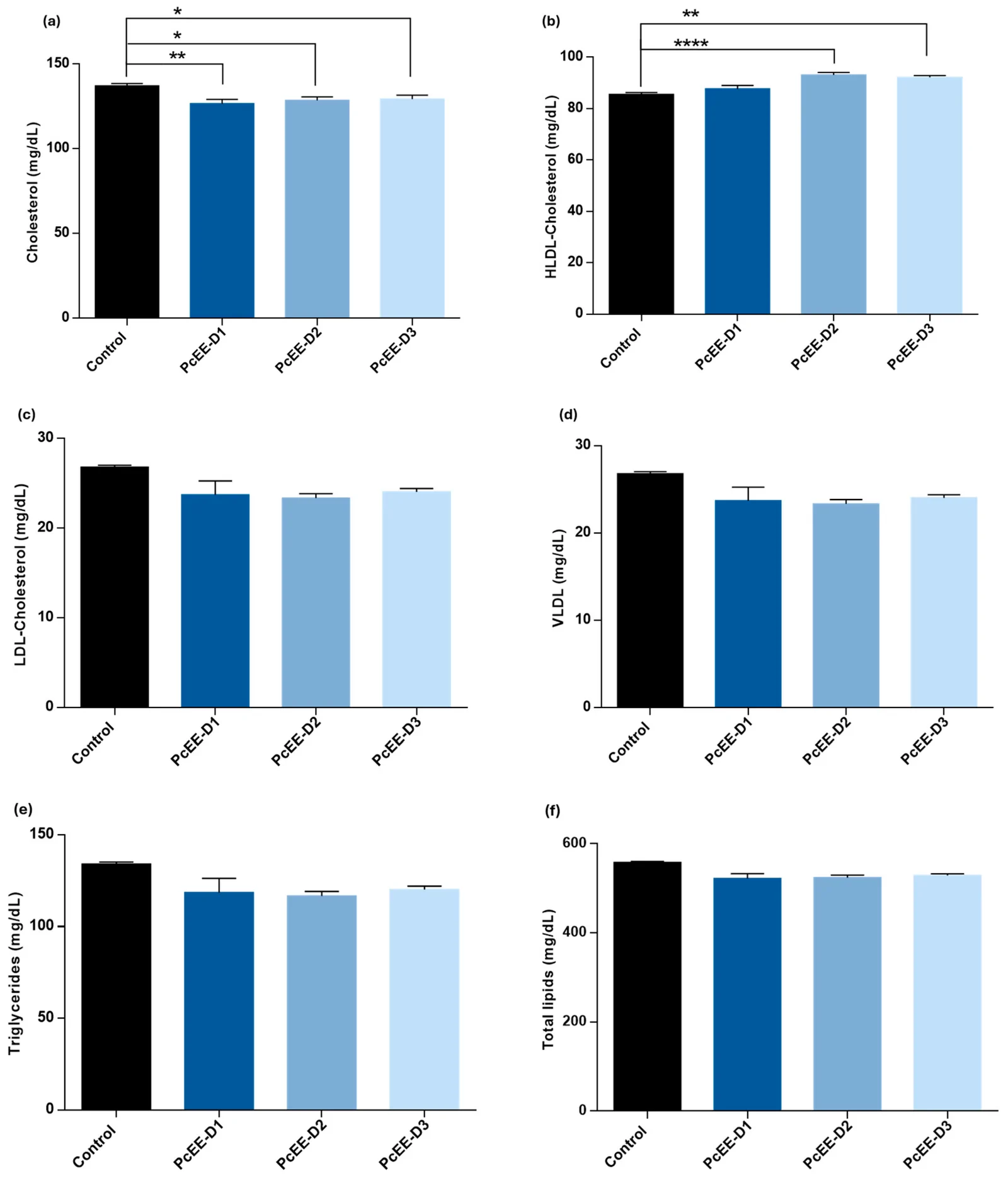
Lipid profile biomarkers in CD-1 mice orally administered the hydroethanolic extract of *P. chevalieri* aerial parts (PcEE) at doses of 170, 600, and 1000 mg/kg for 14 days: (**a**) cholesterol, (**b**) HDL, (**c**) LDL, (**d**) VLDL, (**e**) triglycerides, (**f**) total lipids, and comparison with the control group. Data are expressed as the Mean ± S.E.M., *n* = 6–8. Significance level among different groups: * *p* < 0.05; ** *p* < 0.01; and **** *p* < 0.0001.

The lipid biomarkers studied were total cholesterol, high-density lipoprotein (HDL), low-density lipoprotein (LDL), very-low-density lipoprotein (VLDL), triglycerides, and total lipids. The results for all these biomarkers are exhibited in Figure 5.

The lipid profile revealed that all doses of *P. chevalieri* extract significantly reduced total cholesterol relative to control animals. The greatest reduction occurred at 600 mg/kg, paralleled by a significant increase in HDL–cholesterol. Serum levels of LDL–cholesterol, VLDL, triglycerides, and total lipids were not significantly affected, indicating that the extract modulated cholesterol metabolism without broadly disturbing the other lipid biomarkers.

### 3.3. Genotoxicity Potential (Ames Test)

**Figure 6:**
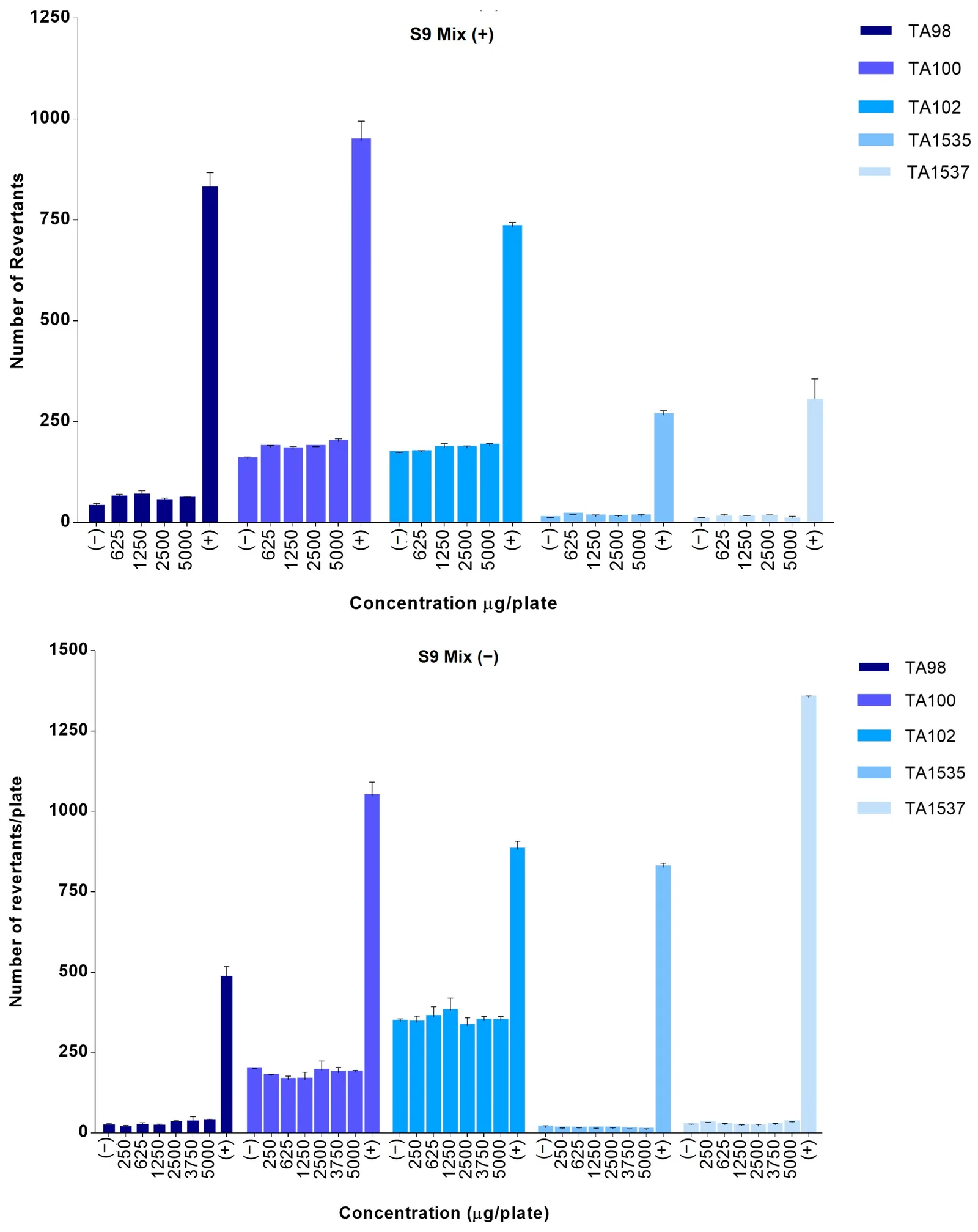
Number of revertant colonies induced by PcEE in the bacterial reverse mutation assay with (S9 mix (+)) and without S9 (S9 mix (−)) metabolic activation. (−), vehicle control; (+), positive control.

Histidine-auxotrophic strains of *Salmonella* Typhimurium TA98, TA100, TA102, TA1535 and TA1537 were used, with (S9 mix (+)) and without (S9 mix (−)) (Figure 6) a metabolic activation system (S9), to assess the genotoxic potential of PcEE.

PcEE did not increase the number of revertant colonies by two-fold (TA98, TA100, and TA102) or three-fold (TA1535, TA1537) or more relative to the negative control, with (S9 mix (+)) and without (S9 mix (−)) metabolic activation. In contrast, the positive controls induced a clear increase in revertant colonies in all tested strains relative to the negative control, confirming the validity of the assay system. Under the conditions tested, PcEE showed no mutagenic activity in the bacterial reverse mutation assay at concentrations up to 5000 μg/plate, with and without metabolic activation.

## 4. Discussion

Based on their long history of use in traditional medicine, medicinal plants have been regarded as safe and natural. However, research on their phytochemical and toxicological profile has shown that a significant number of the plants used in traditional medicine have toxic potential [42].

The toxicological profile of most medicinal plants used in Africa is unknown, and herbal medicines are not held to the same safety and efficacy standards as pharmaceuticals in most countries [43]. In this sense, the WHO and research institutes in several African countries are making significant efforts to highlight the importance of studying plants that have ethnomedical value in Africa [44]. Previous work by our team showed that both an aqueous extract (prepared at 95 °C) and a 70% hydroethanolic extract of *P. chevalieri* aerial parts, which have qualitatively similar chemical profiles, had potential to manage type 2 diabetes, supporting the primary traditional therapeutic use of this plant. These findings may encourage a wider use of *P. chevalieri* in traditional medicine, making the characterization of its safety profile crucial for its safe use as a herbal medicine. The Organization for Economic Cooperation and Development (OECD) and the International Conference on Harmonization (ICH) have established standard guidelines for evaluating the toxicity of chemicals and pharmaceuticals as part of continuing efforts to harmonize toxicity testing at an international level.

In this study, we adopted the recommendations of the OECD and ICH guidelines for oral toxicity and genotoxicity assessments, with modifications to comply with European Medicines Agency (EMA) guidelines for herbal medicinal products, as established by the Committee on Herbal Medicinal Products (HMPC) [33,38,39,40,45].

A standardized *P. chevalieri* aerial parts 70% hydroethanolic extract was used to assess the preclinical safety of *P. chevalieri*. The standardization was based on its phenolic content and chemical profile, characterized mainly by the presence of flavonoids and phenolic acids as the major marker compounds.

The oral toxicity study was performed at doses of 170, 600, and 1000 mg/kg of extract. The extract was administered orally once daily for 14 days to CD-1 mice, and the animals were observed for clinical signs of toxicity and mortality. 

Body weight did not differ significantly between the PcEE-treated groups and the control group, as no significant change (*p* > 0.05) was observed between the initial and final weights of all the animals (treated and control) throughout the experimental procedure. Regarding food intake, while the control group, PcEE-D1-, and PcEE-D2-treated groups showed no significant change in this parameter, the PcEE-D3-treated animals showed reduced food consumption when compared to all other groups. However, the reduced food intake was not characterized as a sign of toxicity, as evidence from several studies shows that decreased appetite in animals treated with herbal formulations may be an adaptive behavior that leads to lower caloric intake by the animals [46].

Oral administration of *P. chevalieri* extracts to mice at doses up to 1000 mg/kg revealed no significant hepatic toxicity, as indicated by unchanged ALT and AST levels. However, a mild elevation in creatinine without a corresponding increase in urea was noted. Although an increase in creatinine levels can be considered a sign of renal injury, the values observed were within reference values (0.2–0.9 mg/dL) [42] and historical control values (0.28–0.37 mg/dL) [34,35,47]. Additionally, minor variations in kidney function, such as the diuretic effect, are transient effects frequently observed when extracts rich in phenolic acids are administered to rodents [34,48]. In this sense, the observed increase in creatinine was not interpreted as an unequivocal sign of toxicity. Nevertheless, to rule out possible subclinical effects on renal function, chronic toxicity studies are further recommended.

Regarding pancreatic function, no adverse changes were observed in amylase levels. Although a significant but isolated rise in lipase at the intermediate dose (600 mg/kg) may indicate minimal pancreatic disturbance, the observed clinical signs, the absence of a dose response, and the macroscopic examination of the pancreas in the necropsy led us to discard possible toxic effects.

Concerning the lipid metabolism, PcEE showed a favorable effect on lipidic biomarkers, as all tested doses were associated with a statistically significant reduction in total cholesterol (*p* < 0.05), with the most pronounced effect observed at 600 mg/kg, which also increased HDL–cholesterol. No alterations in other lipid fractions were observed. These results broadly align with our previous study, where the hydroethanolic extract of *P.chevalieri* also exhibited cholesterol-lowering effects [30]. However, the magnitude of these effects should be confirmed with further studies in specific hypercholesterolemic models to confirm the effect observed in both studies.

Genotoxicity is the most common safety data gap in the safety assessment of herbal medicines [49]. According to the Committee on Herbal Medicinal Products (HMPC), “many herbal preparations contained in well-established or traditional herbal medicinal products have an accepted safety profile, which has been based on their long-term medicinal and/or food use.” [1]. However, as revealed by increasing evidence, relying solely on the history of use of herbal medicines exclusively used in traditional medicine may not be sufficient to address the mutagenic or carcinogenic risk, as these adverse effects are delayed and difficult to track or connect to exposure to herbal medicine [49].

The Ames test performed with *P. chevalieri* hydroethanolic extract evaluated across five *Salmonella* Typhimurium strains (with and without metabolic activation) revealed no mutagenic effects at any concentration tested. The number of revertant colonies consistently remained within the range observed for negative controls, while positive controls validated the assay system. The criteria established by the Ames test protocol and the regulatory guidelines to characterize a substance as genotoxic in at least one of the *Salmonella* Typhimurium strains are an increase in revertant colonies up to 2–3 fold and/or a clear dose-dependent response. Thus, the lack of such findings here supports the extracts’ safety [38,39,40,50].

The bioactive compounds present in herbal medicines have long been associated with their pharmacological and toxicological potential [13,42]. The main marker compounds identified in PcEE were phenolic acids and flavonoids, namely, quercetin glycosides (Table S1). It is well-known that quercetin exhibits a positive response in the Ames test in some strains of *Salmonella* Typhimurium. However, PcEE did not present genotoxic potential in any of the test strains. This result is in line with several studies that have shown that the genotoxicity of one compound may not represent the toxicity of the whole extract, as a synergistic effect of other compounds in the extract may modulate this effect. Additionally, it has also been shown that the glycosylation of quercetin tends to diminish the pro-oxidant activity of quercetin, resulting in less mutagenicity [51].

The obtained results are the first to characterize the preclinical safety profile of *P. chevalieri.* Nevertheless, mild creatinine elevations and pancreatic enzyme alterations observed point to the need for additional toxicity studies, namely a 28-day repeated oral toxicity study and a 90-day chronic toxicity study, as well as a mechanistic exploration. The lack of overall toxicity and genotoxic potential of *P. chevalieri* is an important contribution to the safety profile of *P. chevalieri* as an herbal medicine.

## 5. Conclusions

The oral toxicity and genotoxicity of *P. chevalieri* hydroethanolic extract were assessed. Under the conditions tested, PcEE showed no oral toxicity in CD-1 mice and no genotoxicity with and without metabolic activation. These findings support the safety of this extract and are an important positive step towards validating the use of *P. chevalieri* as a herbal medicine. However, long-term in vivo studies, along with further pharmacological and toxicological studies, are needed to fully characterize its medicinal properties and toxicological profile.

## Data Availability

The data is contained within the article and Supplementary Material.

## Supplementary Materials

The following supporting information can be downloaded at: https://www.mdpi.com/article/10.3390/jox16060198/s1; Table S1: Major marker secondary metabolites identified by HPLC-ESI/MS/MS in *P. chevalieri* 70% hydroethanolic extract; Table S2: Clinical signs and behavioral patterns of mice treated with vehicle control and *P. chevalieri* extract.

## Abbreviations

The following abbreviations are used in this manuscript:

ALT, alanine aminotransferase; AST, aspartate aminotransferase; bw, body weight, DER, drug extract ratio, EMA, European Medicines Agency; GAE, gallic acid equivalents; HDL, high-density lipoprotein; HPLC, high-performance liquid chromatography; ICH, International Conference on Harmonization; LDL, low-density lipoprotein; MS, mass spectrometry; OECD, Organization for Economic Cooperation and Development; PcEE, *Periploca chevalieri* hydroethanolic extract; PcEE-D1, *Periploca chevalieri* hydroethanolic extract dose 1, PcEE-D2, *Periploca chevalieri* hydroethanolic extract dose 2, PcEE-D3, *Periploca chevalieri* hydroethanolic extract dose 3; S.D. Standard deviation; S.E.M., Standard Error of the Mean; VLDL, very-low-density lipoprotein.
