# Supplementary material for: Safety Assessment of a Periploca chevalieri Browicz Standardized Extract: Oral Toxicity and Genotoxicity Studies
Source: J Xenobiot. 2026 Oct 16;16(6):198. doi: 10.3390/jox16060198 (PMC13706384; doi:10.3390/jox16060198)
Supplement: Supplementary file 1 [file jox-16-00198-s001.zip › jox-4576632-supplementary.pdf]

Table S1. Major marker secondary metabolites identified by HPLC-ESI/MS/MS in *P. chevalieri* 70% hydroethanolic extract

Peak	t_R	$[M^-H]^-$	MS/MS *m/z* (Relative Abundance)	Tentative Identification
1	6.2	191	191; 127; 93; 85	Quinic acid
2	8.5	305	167; 139; 137; 125	(+)- Gallocatechin
3	9.6	593	425; 407; 289; 125	Proanthocyanidin B (Dimer)
4	12.2	593	451; 407; 425; 289; 125	Proanthocyanidin B (Dimer)
5	13.2	897	593; 305; 289; 125	Proanthocyanidin B (Trimer)
6	18.3	353	191; 179; 161; 135	3-*O*-caffeoyilquinic acid[1] (neochlorogenic acid)
7	19.2	577	425; 407; 289; 125	Proanthocyanidin B (Dimer)
8	20.8	577	425; 407; 289; 125	Proanthocyanidin B (Dimer)
9	21.6	881	593; 577; 305; 289	Proanthocyanidin B (Trimer)
10	22.1	305	167; 139; 137; 125	(-)-Epigallocatechin
11	24.8	289	289; 203; 125; 109	(+)- Catechin[1]
12	28.9	353	191; 179; 173: 135	4-*O*-caffeoyilquinic acid (cryptochlorogenic acid)
13	30.7	353	191; 179; 173: 161	5-*O*-caffeoyilquinic acid[1] (chlorogenic acid)
14	35.8	289	289; 151; 149; 125; 109	(-)-Epicatechin[1]
15	47.4	755	755; 609; 463; 301	Quercetin 3-*O*-(2″,6″-di-*O*-rhamnosyl) galactoside
16	47.6	755	755; 609; 463; 301	Quercetin 3-*O*-(2″,6″-di-*O*-rhamnosyl) glucoside
17	50.3	609	609; 301; 300	Quercetin-3-*O*-rutinoside[1] (rutin)
18	50.3	477	301; 300; 179; 151	Quercetin-3-*O*-glucoronide
19	50.3	463	301; 300; 271; 151	Quercetin-3-*O*-galactoside[1] (hyperoside)
20	50.8	515	353; 191; 179; 173	1,4 dicafeoyilquinic acid

[1]Compounds identified with commercial reference standard by co-chromatography; MS-MS—mass spectrometry; *m/z*—mass-to-charge ratio; t_R—retention time.

Table S2. Clinical signs and behavioral patterns of mice treated with vehicle control and *P. chevalieri* extract.

Time		Parameters						
		[1.]Lesions, changes in fur/skin (no. animals)	[2.]Changes in eyes and mucous membrane (no. animals)	[3.]Presence of excessive grooming, repetitive circling	[4.]Autonomic activity* (no. animals)	[5.]Changes in posture and response to handling (no. animals)	[6.]Ocurrence of secretions and excrecions	[7.] Mortality (no. animals)
**24h**	CG	A	A	A	A	A	A	A
	D1	A	A	A	A	A	A	A
	D2	A	A	A	A	A	A	A
	D3	A	A	A	A	A	A	A
**48h**	CG	A	A	A	A	A	A	A
	D1	A	A	A	A	A	A	A
	D2	A	A	A	A	A	A	A
	D3	A	A	A	A	A	A	A
**5 days**	CG	**P (1)**	A	A	A	A	A	A
	D1	**P (2)**	A	A	A	A	A	A
	D2	**P (2)**	A	A	**P (2)**	A	A	A
	D3	A	A	A	A	A	**P**	A
**10 days**	CG	A	A	A	A	A	A	A
	D1	A	A	A	A	A	A	A
	D2	A	A	A	A	A	A	A
	D3	A	A	A	A	A	A	A
**14 days**	CG	A	A	A	A	A	**P**	A
	D1	A	A	A	A	A	A	**P (1)**
	D2	A	A	A	A	A	A	**P (1)**
	D3	A	A	A	A	A	**P**	**P (2)**

Abbreviations: CG- control group; D1 (Dose 1- 50mg/kg); D2 (Dose 2- 400mg/kg); D3 (Dose 3- 1000mg/kg); P- Present; A- Absent; * Autonomic activity: lacrimation, piloerection, unusual respiration; **Number of animals affected between parentheses.

[1.] On day 6 one animal from control group showed a small lesion on the paw. On day 8, two animals from D2 group showed lesions in the skin, both had small wounds in the back with loss of fur; In D3 group two animals presented lesions, one had a wounded paw with dry blood, the other animal presented a small wound in the chest.
[2.] No observations.
[3.] No observations.
[4.] On day 9 two animals in D2 group presented with unsual respiration.
[5.] No observations.
[6.] After 3 days the feces of the animals in D3 group started to show a yellowish color beside normal color feces.
[7.] On day 11 one animal from D1 group died immediately after the the extract administration. On day 13, two animals from D3 group died immediately after the extract administration. On day 14 one animal from D2 group died after also immediately after the extract administration. The deaths were interpreted as gavage-related administration errors.
